# Emerging Invasive Group A *Streptococcus* M1_UK_ Lineage Detected by Allele-Specific PCR, England, 2020^1^

**DOI:** 10.3201/eid2905.221887

**Published:** 2023-05

**Authors:** Xiangyun Zhi, Ho Kwong Li, Hanqi Li, Zuzanna Loboda, Samson Charles, Ana Vieira, Kristin Huse, Elita Jauneikaite, Lucy Reeves, Kai Yi Mok, Juliana Coelho, Theresa Lamagni, Shiranee Sriskandan

**Affiliations:** Imperial College London, London, UK (X. Zhi, H.K. Li, H. Li, Z. Loboda, S. Charles, A. Vieira, K. Huse, E. Jauneikaite, L. Reeves, K.Y. Mok, S. Sriskandan);; United Kingdom Health Security Agency, London (J. Coelho, T. Lamagni)

**Keywords:** *Streptococcus pyogenes*, M1_UK_, emm1, scarlet fever, molecular diagnostics, PCR, bacteria, respiratory infections, streptococci, superantigen, allele-specific, emerging lineage, England, iGAS, group A *Streptococcus*

## Abstract

Increasing reports of invasive *Streptococcus pyogenes* infections mandate surveillance for toxigenic lineage M1_UK_. An allele-specific PCR was developed to distinguish M1_UK_ from other *emm*1 strains. The M1_UK_ lineage represented 91% of invasive *emm*1 isolates in England in 2020. Allele-specific PCR will permit surveillance for M1_UK_ without need for genome sequencing.

Upsurges in invasive group A *Streptococcus* (GAS) infections have been widely reported in England and elsewhere (*1*), emphasizing the need to examine the relationship between circulating *S. pyogenes* that cause pharyngitis and scarlet fever and cases of invasive disease. Although many factors, such as exposure history, underlying conditions, viral co-infection, and genetic susceptibility, might increase susceptibility to *S.* *pyogenes* infection, strain-specific virulence might also be crucial.

In England, where both scarlet fever and invasive *S. pyogenes* infections are notifiable, pronounced upsurges in scarlet fever were recorded over an 8-year period (*2*,*3*), but subsided during the COVID-19 pandemic. During the 2015–16 season, a notable increase in invasive infections was observed that had not been evident previously (*4*). Both scarlet fever and invasive infections were associated with the emergence of M1_UK_, a new sublineage of *emm*1 *S. pyogenes* (*4*) that appeared to outcompete the highly successful, contemporary epidemic *emm*1 M1_global_ strain, which emerged and spread globally during the 1980s (*5*,*6*). Despite an unchanged phage repertoire, M1_UK_ strains produce more superantigenic scarlet fever toxin SpeA (streptococcal pyrogenic exotoxin A) than contemporary M1_global_ *S. pyogenes* strains (*4*).

*emm*1 *S. pyogenes* strains are highly virulent (*5*) and disproportionately associated with invasive infections; any increase in the prevalence of *emm*1 strains in persons with pharyngitis or scarlet fever is, therefore, a public health concern. Known distribution of M1_UK_ is largely limited to those countries undertaking and reporting genome sequencing (Figure 1). M1_UK_ has been identified in other countries in Europe, from a single isolate in Denmark (*4*) to dominant status in the Netherlands (*7*). The lineage has also been reported in North America; the Public Health Agency of Canada reported that 17/178 (10%) of *emm*1 isolates from 2016 were M1_UK_ (*8*). This finding contrasts with a reported M1_UK_ frequency of just 0%–2.8% of *emm*1 isolates in the United States, according to the Active Bacterial Core surveillance system of the US Centers for Disease Control and Prevention; however, the low US frequency was associated with severe infections (*9*). Of note, most reports used genomic data that were >5 years old, so a reappraisal of prevalence is needed. A recent study in Australia using data through 2020 indicated expansion of M1_UK_ in Queensland and Victoria (*10*). The authors identified acquisition of an additional phage encoding superantigen genes *ssa* and *spec* and a single-nucleotide polymorphism (SNP) implicated in SpeA upregulation in the M1_UK_ lineage. Multicountry increases in GAS infections (*1*) since pandemic restrictions were lifted underscore the importance of increasing global surveillance of lineages that have potentially enhanced fitness, such as M1_UK_.

**Figure 1:**
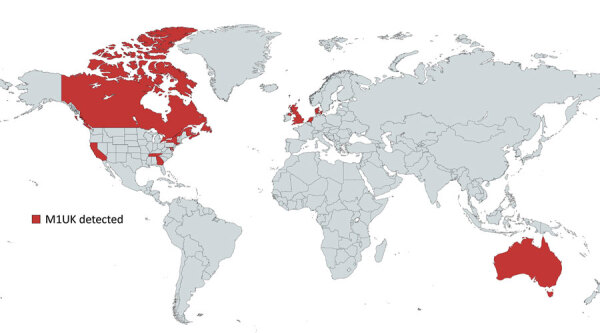
Countries and US states with reported M1_UK_ *Streptococcus* *pyogenes* cases. Map created by using MapChart (https://www.mapchart.net) as part of a study of emerging invasive group A *Streptococcus* M1_UK_ lineage detected by allele-specific PCR, England, 2020.

## The Study

Genetic distinction between M1_UK_ and M1_global_ strains is possible by using whole-genome sequencing to detect the 27 SNPs that characterize the M1_UK_ lineage (*4*), but sequencing technology is not available in all countries. We designed an allele-specific PCR (AS-PCR) method to detect M1_UK_-specific SNPs in the *rofA*, *gldA*, and *pstB* genes. We chose amplification targets to separate M1_UK_ and M1_global_ strains but also to identify strains from less common intermediate sublineages that had only 13 or 23 of the 27 M1_UK_-specific SNPs (*4*). We optimized PCR conditions for each pair of amplicons by using DNA from control strains for each lineage (Table; Appendix Figure). Collecting bacterial samples from patients was part of routine clinical care; collecting surplus samples after anonymizing patient information was approved by the West London National Research Ethics Committee (approval no. 06/Q0406/20).

**Table:** PCR primers and conditions used to differentiate M1_global_ and M1_UK_ *Streptococcus pyogenes* lineages in study of emerging invasive group A *Streptococcus* M1_UK_ lineage detected by allele-specific PCR, England, 2020*

Target gene	Primer type†	Sequences‡	PCR cycle conditions	Product, bp
*rofA*	WT sequence	TGTTAATTGCTTGGTTAAATCA	30 cycles of 95°C for 3 min, 45 s; 59.2°C for 30 s; 72°C for 1 min (final cycle: 5 min)	278
	Forward-SNP	5′-TGTTAATTGCTTGGTTAAAG**t**A-'3		
	Forward-WT	5′-TGTTAATTGCTTGGTTAAAGCA-'3		
	Reverse	5′-GCTCATCTCCTAACGGATTCTT-'3		
*gldA*	WT sequence	AGATGGGTTAGCAACATGG	30 cycles of 95°C for 3 min, 45 s; 61.8°C for 30 s;72°C for 1 min (final cycle: 5 min)	292
	Forward-SNP	5′-AGATGGGTTAGCAACAA**a**G-'3		
	Forward-WT	5′-AGATGGGTTAGCAACAAGG-'3		
	Reverse	5′-GAATAGCACCTGTCAGCG-'3		
*pstB*	WT sequence	GATAAATCAATCTTAGACCA	30 cycles of 95°C for 3 min, 45 s; 50°C for 30 s; 72°C for 1 min (final cycle: 5 min)	287
	Forward-SNP	5′-GATAAATCAATCTTAGAT**a**A-'3		
	Forward-WT	5′-GATAAATCAATCTTAGATCA-'3		
	Reverse	5′-CGTGAGGCTTGCTGCATTGAG-'3		

*SNP, single-nucleotide polymorphism; WT, wild-type. †Forward primers were designed to detect either the targeted SNP (M1_UK_) or WT (M1_global_) sequences. ‡Lowercase bold letters in primer sequences denote the base complementary to the targeted SNP in the M1_UK_ sequence. Underlined uppercase letters indicate an additional mismatched base introduced into primer sequences to increase primer specificity.

To evaluate allele-specific PCR, we tested whether the *rofA* and *pstB* primers correctly identified lineages of 27 newly genome-sequenced noninvasive *emm*1 *S. pyogenes* strains isolated during 2017–18 and collected by the infection bioresource at Imperial College. We artificially enriched the isolates for M1_global_ strains to ensure adequate numbers of each lineage: 8/27 isolates were M1_global_, and 19/27 were M1_UK_. PCR amplification of *rofA* and *pstB* alleles from those isolates assigned 100% of strains to the correct lineage previously identified by sequencing (Appendix Table 1).

To evaluate the ability of AS-PCR to identify *emm*1 isolates from M1_global_, M1_UK_, and intermediate sublineages (*4*), we tested 16 strains from 2013–2016 that comprised 4 isolates each of M1_global_, M1_13snps_, M1_23snps_, and M1_UK_ lineages (Appendix Table 2). SNPs were correctly detected in the *rofA* gene from all M1_13snps_, M1_23snps_, and M1_UK_ isolates (Appendix Table 3). SNPs were also correctly detected in *gldA* from all M1_23snps_ and M1_UK_ isolates but not M1_global_ or M1_13snps_ isolates_._ Finally, SNPs in *pstB* were only identified in M1_UK_ isolates_._ Thus, in all cases, SNP profiles determined by AS-PCR were consistent with strain-specific genome sequences.

In England, submission of all isolates from invasive infection is requested by the UK Health Security Agency reference laboratory for *emm* genotyping. *emm*1 isolates are routinely the dominant genotype among invasive sterile-site isolates, typically representing 20%–30% of invasive infections. During 2020, when incidence of common respiratory infections was reduced by COVID-19–related public health interventions, *emm*1 *S. pyogenes* frequency varied each month from 0%–24% of all invasive infections and decreased toward the end of the year. We subjected all 305 invasive *emm*1 *S. pyogenes* isolates from 2020 that were available for this study to AS-PCR (Appendix Table 4). AS-PCR identified M1_UK_-specific SNPs in *rofA*, *gldA*, and *pstB* in 278/305 (91.1%) of isolates, which were, therefore, assigned to the M1_UK_ lineage. No SNPs were detected in the remaining 27 isolates, which were assigned to M1_global_; no intermediate lineage *emm*1 strains were identified in isolates collected during 2020 by using AS-PCR.

We performed Western blot analysis of 10 M1_UK_ isolates identified by AS-PCR. We confirmed that SpeA production was similar to M1_UK_ strains tested previously; however, we did not quantify SpeA production.

## Conclusions

**Figure 2:**
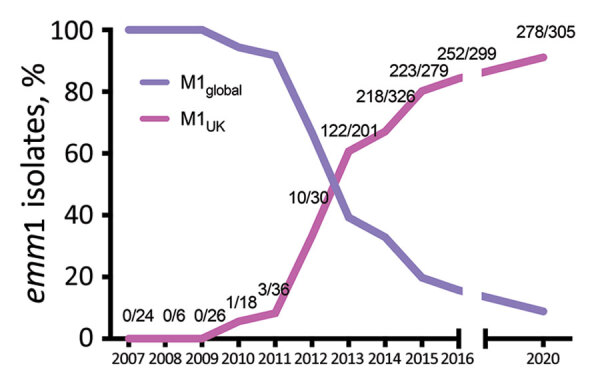
Prevalence of M1_UK_ and M1_global_ *Streptococcus* *pyogenes* lineages over time in study of emerging invasive group A *Streptococcus* M1_UK_ lineage detected by allele-specific PCR, England, 2020. We determined percentages of *emm*1 isolates in England that belonged to M1_UK_ or M1_global_ lineages by using all available *emm*1 *S*. *pyogenes* genome sequences for 2007–2016 (*4*) and all available invasive isolates from 2020 that we tested by allele-specific PCR. Numbers on graph indicate number of isolates assigned as M1_UK_/total number sequenced for each year. Graph was adapted and updated from data previously described (*4*).

The longevity of emergent *S. pyogenes* lineages in a population is difficult to predict. Although an *emm*89_emergent_ acapsular lineage has disseminated globally (*11*), an emergent *emm*3 SpeC-producing lineage, associated with upsurges in scarlet fever and invasive infections, ceased to be detectable within a few years (*12*). Taken together with previously reported genome-sequenced *emm*1 isolates (Figure 2), AS-PCR results indicated that the M1_UK_ lineage continued to expand among invasive *S. pyogenes* isolates from 2016 to the end of 2020 in England.

Increased invasive GAS activity in several countries (*1*) indicates a need for ongoing surveillance of novel lineages, given the potential public health effects. AS-PCR provides a readily available method to detect M1_UK_ that is straightforward and, for screening purposes only, can be simplified by using only *rofA* primers to identify M1_UK_ or associated sublineages. A limitation of our study is that the assay requires validation in reference laboratory settings. AS-PCR does not replace genome sequencing as the preferred method for surveillance of highly pathogenic bacteria, but sequencing is not widely available and is expensive.

*emm*1 strains have accounted for >50% of invasive infections in children in England during the 2022–23 season (*13*). Our results indicate that the M1_UK_ lineage remained dominant in England and expanded to the end of 2020, and contact tracing in 2018 demonstrated a high frequency of secondary acquisition of M1_UK_ in school outbreak settings (*14*). Given the recognized association between *emm*1 *S. pyogenes* and fatal outcome of invasive infections (*15*), enhanced surveillance for the M1_UK_ sublineage is warranted. We conclude that AS-PCR is a readily available method to determine whether *emm*1 *S*. *pyogenes* isolates belong to the M1_UK_ clade without need for genome sequencing and will improve surveillance of invasive GAS strains.

Appendix Additional information for emerging invasive group A *Streptococcus* M1_UK_ lineage detected by allele-specific PCR, England, 2020.
