# Supplementary material for: Emerging Invasive Group A Streptococcus M1UK Lineage Detected by Allele-Specific PCR, England, 2020
Source: Emerg Infect Dis. 2023 May;29(5):1007–10. doi: 10.3201/eid2905.221887 (PMC10124639; doi:10.3201/eid2905.221887)
Supplement: Appendix — Additional information for emerging invasive group A Streptococcus M1UK lineage detected by allele-specific PCR, England, 2020. [file 22-1887-Techapp-s1.pdf]

Article DOI: https://doi.org/10.3201/eid2905.221887
EID cannot ensure accessibility for supplementary materials supplied by authors. Readers who have difficulty accessing supplementary content should contact the authors for assistance.

Emerging Invasive Group A *Streptococcus* $M1_{UK}$ Lineage Detected by Allele-Specific PCR, England, 2020

Appendix

Appendix Table 1. Evaluation of *rofA* and *pstB* allele-specific primers for lineage assignment by using genome-sequenced *emm*1 strains from 2017–2018*

Year	Lineage†	Total no. strains	No. strains yielding PCR products with allele-specific primer pairs‡			
			rofA, $M1_{global}$	*rofA*, $M1_{UK}$	*pstB*, $M1_{global}$	*pstB*, $M1_{UK}$
2017	$M1_{global}$	3	3	0	3	0
	$M1_{UK}$	6	0	6	0	6
2018	$M1_{global}$	5	5	0	5	0
	$M1_{UK}$	13	0	13	0	13

*Allele-specific PCR results performed on extracted DNA were consistent with whole-genome sequencing of DNA from 27 *emm*1 noninvasive *S. pyogenes* isolates (n = 9 from 2017, n = 18 from 2018, enriched for $M1_{global}$ strains). Sequences for *emm*1 test strains were submitted to the European Nucleotide Archive (https://www.ebi.ac.uk/ena) under project no. PRJEB58440.
†Lineage was assigned on the basis of whole-genome sequencing results.
‡Primer pairs for *rofA* and *pstB* genes that detect $M1_{global}$ and $M1_{UK}$ lineages are listed in the Table (main text).

Appendix Table 2. Accession numbers of genome-sequenced strains used to validate allele-specific PCR used to detect all sublineages*

M1 lineage	Year	Accession no.	Sample ID
$M1_{13snps}$	2013	ERS1594714	PHEGAS005
$M1_{13snps}$	2013	ERS1594852	PHEGAS127
$M1_{13snps}$	2015	ERR1733723	GASEMM2799
$M1_{13snps}$	2015	ERR1734520	GASEMM2970
$M1_{23snps}$	2013	ERS1594734	PHEGAS025
$M1_{23snps}$	2013	ERS1594744	PHEGAS035
$M1_{23snps}$	2013	ERS1594757	PHEGAS048
$M1_{23snps}$	2013	ERS1594864	PHEGAS137
$M1_{global}$	2013	ERS1594798	PHEGAS168
$M1_{global}$	2013	ERS1594822	PHEGAS097
$M1_{global}$	2014	ERR1732733	GASEMM1027
$M1_{global}$	2015	ERR1734897	GASEMM2755
$M1_{UK}$	2013	ERS1594722	PHEGAS013
$M1_{UK}$	2014	ERR1733140	GASEMM0629
$M1_{UK}$	2015	ERR1733678	GASEMM3027
$M1_{UK}$	2016	ERS1594947	PHEGAS285

*Sequences from European Nucleotide Archive (https://www.ebi.ac.uk/ena) were previously listed in reference (*4*) (main text).

Appendix Table 3 Validation of *rofA*, *pstB*, and *gldA* allele-specific primers for lineage assignment by using genome-sequenced *emm*1 strains, including intermediate sublineages*

Lineage†	Total no. strains	No. strains yielding PCR products with allele-specific primer pairs‡					
		rofA, $M1_{global}$	rofA, $M1_{UK}$	gldA, $M1_{global}$	gldA, $M1_{UK}$	pstB, $M1_{global}$	pstB, $M1_{UK}$
$M1_{global}$	4	4	0	4	0	4	0
$M1_{13snps}$	4	0	4	4	0	4	0
$M1_{23snps}$	4	0	4	0	4	4	0
$M1_{UK}$	4	0	4	0	4	0	4

*Whole-genome sequenced *S. pyogenes emm*1 strains from 2013 and 2014 (n = 16, accession nos. in Appendix Table 2) were analyzed by using *rofA*, *gldA*, and *pstB* allele-specific PCR primers. Strains were selected for the presence of 0/27, 13/27, 23/27, or 27/27 single nucleotide polymorphisms specific for $M1_{UK}$ (4 isolates of each). Allele-specific PCR results were consistent with whole-genome sequencing.
†Lineage was assigned on the basis of whole-genome sequencing results.
‡Primer pairs for *rofA*, *gldA*, and *pstB* genes that detect $M1_{global}$ and $M1_{UK}$ lineages are listed in the Table (main text).

Appendix Table 4. Use of allele-specific PCR to test 305 invasive *emm*1 *Streptococcus pyogenes* isolates of unknown lineage that were collected in 2020*

	No. strains yielding PCR products with allele-specific primer pairs‡						
Lineage†	*rofA*, $M1_{global}$	*rofA*, $M1_{UK}$	*gldA*, $M1_{global}$	*gldA*, $M1_{UK}$	*pstB*, $M1_{global}$	*pstB*, $M1_{UK}$	Total
$M1_{global}$	27	0	27	0	27	0	27
$M1_{UK}$	0	278	0	278	0	278	278

*All invasive *emm*1 strains (n = 305) submitted to the reference laboratory for *emm* genotyping and identified as *emm*1 were evaluated by allele-specific PCR. A total of 278 isolates yielded PCR products consistent with the $M1_{UK}$ lineage. No intermediate isolates were identified.
†Lineage was inferred by results of allele-specific PCR.
‡Primer pairs for *rofA*, *gldA*, and *pstB* genes that detect $M1_{global}$ and $M1_{UK}$ lineages are listed in the Table (main text).

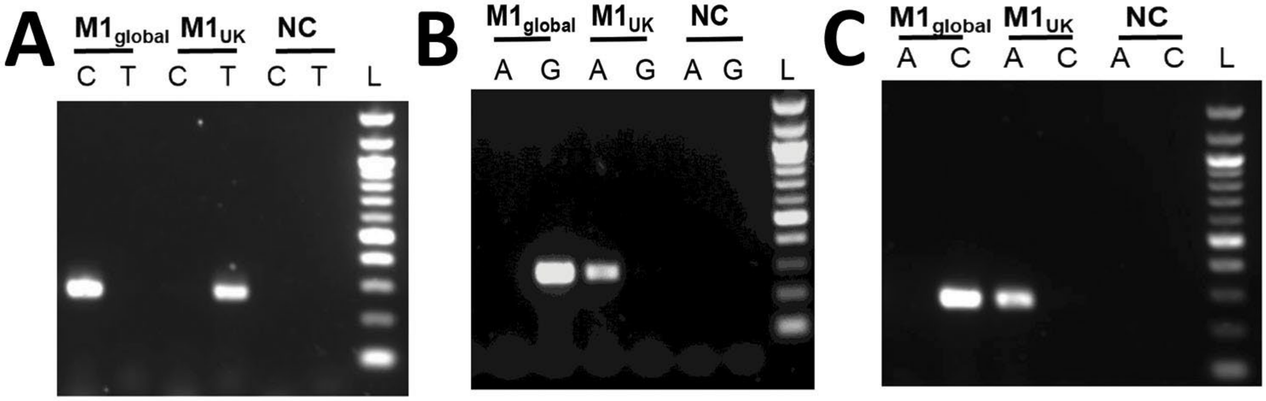

Appendix Figure. Gel electrophoresis demonstrating allele-specific PCR to distinguish between $M1_{global}$ and $M1_{UK}$ *Streptococcus pyogenes* lineages. PCR products after amplification of DNA from $M1_{global}$ and $M1_{UK}$ strains of *S. pyogenes* are shown. (A) *rofA* SNP primers; C and T denote nucleotide differences used in *rofA* forward primers. (B) *gldA* SNP primers; A and G denote nucleotide differences used in *gldA* A or G forward primers. (C) *pstB* SNP primers; A and C denote nucleotides used in *pstB* A or C forward primers. Primer pairs for *rofA*, *gldA*, and *pstB* genes that detect $M1_{global}$ and $M1_{UK}$ lineages are listed in the Table (main text). DNA was isolated from control strains BHS0151 ($M1_{global}$) and BHS581 ($M1_{UK}$) that have been previously described (*3*, main text) and then sequenced. Genome sequences are in the European Nucleotide Archive (https://www.ebi.ac.uk/ena) under accession nos. ERS1020136 ($M1_{global}$) and ERS1020603 ($M1_{UK}$). L, 100 bp DNA ladder; NC, negative control.
